# Validation of a Commercially Available IMU-Based System Against an Optoelectronic System for Full-Body Motor Tasks

**DOI:** 10.3390/s25123736

**Published:** 2025-06-14

**Authors:** Giacomo Villa, Serena Cerfoglio, Alessandro Bonfiglio, Paolo Capodaglio, Manuela Galli, Veronica Cimolin

**Affiliations:** 1 Euleria srl Società Benefit, Via delle Zigherane, 4/A, 38068 Rovereto, Italy; giacomo.villa@euleria.it (G.V.); alessandro.bonfiglio@euleria.it (A.B.); 2 Department of Electronics, Information and Bioengineering (DEIB), Politecnico di Milano, 20133 Milan, Italy; serena.cerfoglio@polimi.it (S.C.); manuela.galli@polimi.it (M.G.); 3 Istituto Auxologico Italiano, IRCCS, S. Giuseppe Hospital, 28824 Piancavallo, Italy; 4 Department of Information Engineering and Computer Science (DISI), University of Trento, 38121 Trento, Italy; 5 Energy Efficient Embedded Digital Architectures (E3DA), Fondazione Bruno Kessler, 38121 Trento, Italy; 6 Department of Biomedical, Surgical and Dental Sciences, University of Milan, 20133 Milan, Italy; p.capodaglio@auxologico.it; 7 UOC Musculoskeletal and Metabolic Rehabilitation, IRCCS Istituto Auxologico Italiano, 20133 Milan, Italy

**Keywords:** inertial measurement unit, kinematic analysis, range of motion, validation study, rehabilitation technology

## Abstract

**Highlights:**

**What are the main findings?**

- The IMU-based system demonstrated high concurrent validity with a gold-standard optoelectronic motion capture system across a wide variety of full-body tasks.
- The IMU-based system achieved strong correlations (*r* ≥ 0.77), low RMSE values (generally < 7°), and negligible systematic biases (≤3.9°) compared to the optical reference.

**What are the implications of the main findings?**

- The tested IMU-based system provides clinically acceptable range-of-motion estimates and can serve as a reliable alternative to laboratory-based motion analysis tools.
- Its portability and ease of use make it particularly suited for in-clinic assessments and home-based rehabilitation programs, supporting remote patient monitoring.

**Abstract:**

Inertial measurement units (IMUs) have gained popularity as portable and cost-effective alternatives to optoelectronic motion capture systems for assessing joint kinematics. This study aimed to validate a commercially available multi-sensor IMU-based system against a laboratory-grade motion capture system across lower limb, trunk, and upper limb movements. Fifteen healthy participants performed a battery of single- and multi-joint tasks while motion data were simultaneously recorded by both systems. Range of motion (ROM) values were extracted from the two systems and compared. The IMU-based system demonstrated high concurrent validity, with non-significant differences in most tasks, root mean square error values generally below 7°, percentage of similarity greater than 97%, and strong correlations (*r* ≥ 0.77) with the reference system. Systematic biases were trivial (≤3.9°), and limits of agreement remained within clinically acceptable thresholds. The findings indicate that the tested IMU-based system provides ROM estimates statistically and clinically comparable to those obtained with optical reference systems. Given its portability, ease of use, and affordability, the IMU-based system presents a promising solution for motion analysis in both clinical and remote rehabilitation contexts, although future research should extend validation to pathological populations and longer monitoring periods.

## 1. Introduction

Motor rehabilitation plays a crucial role in restoring functional abilities in individuals affected by injuries, acute or chronic diseases, and other musculoskeletal or neurological conditions [1]. It encompasses targeted physical therapies designed to enhance motor coordination, promote neuroplasticity, improve strength, and prevent muscle atrophy, ultimately contributing to improved quality of life [2,3,4]. Given that motor impairments can lead to varying degrees of functional limitations, the precise and objective quantification of joint range of motion (ROM) is fundamental for assessing functional deficits and evaluating the effectiveness of rehabilitation interventions. ROM is defined as the angular displacement of a joint from its initial position to its maximum movement in a specific direction and is traditionally measured using a manual goniometer.

Manual goniometers are widely used in clinical settings due to their simplicity, cost-effectiveness, and ability to provide direct joint ROM measurements [5]. However, their accuracy is influenced by multiple factors, including the examiner’s expertise and the specific joint assessed [6,7]. Inter-rater reliability also varies, generally showing higher consistency in the upper limbs compared to the lower limbs [8]. Despite their practicality, goniometers are limited to measuring static joint angles in a single plane, making them unsuitable for dynamic motion assessments [9]. Consequently, advanced motion capture technologies have emerged to offer comprehensive assessment of joint kinematics. Among these, optoelectronic marker-based motion capture (MCap) systems have become the gold standard for evaluating body kinematics, providing highly accurate and objective movement analysis [10,11,12]. Nonetheless, their high cost, lack of portability, and dependency on specialized laboratory setup limit their widespread adoption [13].

In this context, the development of inertial measurement units (IMUs) has introduced new possibilities for human motor assessment, significantly increasing their popularity in motion capture studies [14,15,16,17]. IMUs are compact, stand-alone devices integrating data from three-axial accelerometers, gyroscopes, and magnetometers through sensor fusion algorithms and biomechanical models, estimating body segment kinematics in three-dimensional space [18,19,20]. Compared to MCap systems, IMUs significantly expand clinical and research applications due to their portability, cost-effectiveness, and ease of use. Furthermore, their independence from external cameras or laboratory setups makes them ideal for in-home or remote rehabilitation where continuous real-time monitoring is crucial [21,22]. Nonetheless, robust calibration and validation protocols are critical: improper sensor placement, ferromagnetic disturbances [23], and drift errors due to signal integration [24,25] may cause cumulative measurement inaccuracies. Therefore, standardized calibration procedures, involving known reference poses or synchronized movements, are essential to minimize drift and enhance accuracy before clinical application [23,24,25,26].

Over the past decade, IMU-based systems have increasingly been adopted to estimate kinematics across various tasks, including gait analysis [27,28,29], dynamic movements such as jumps [30,31,32] and squats [33,34,35], and lifting tasks [36,37,38]. Despite their good concurrent validity compared with traditional MCap systems, IMU-based joint kinematics require further validation. Specifically, tasks involving large ROMs, abrupt directional changes, or multi-joint coordination can exacerbate sensor fusion errors, underscoring the importance of carefully designed validation studies for different movement patterns [39,40,41].

Several studies have examined the accuracy of IMU-based systems with respect to traditional MCap. Cutti et al. reported root mean square error (RMSE) values below 3.6 degrees and a correlation coefficient of 0.99 in scapulothoracic and humerothoracic kinematics [42]. Similar findings were reported by Parel et al. and Friesen et al. in scapular motions during dynamic tests [43,44]. Regarding elbow kinematics, both Cutti et al. and Fang et al. demonstrated reliable IMU performance in controlled settings and in everyday tasks [42,45]. However, IMUs’ accuracy in assessing upper limb kinematics may vary depending on joint complexity and context of movement [45,46,47]. Regarding lower limb and trunk tasks, Cerfoglio et al. validated a remote IMU system, finding average ROM errors below 5.0 degrees in hip movements and squats [48]. Similarly, Leardini et al. reported average discrepancies below 5 degrees for knee flexion and 3 degrees for chest inclination [49]. IMU-based systems have also demonstrated their effectiveness for inpatient assessment [15,50] and remote home-based rehabilitation [49,51], enabling automated, objective, and real-time evaluations [52,53].

However, while IMU-based systems are generally reported to provide reliable descriptions, their accuracy in estimating joint kinematics can vary depending on system complexity and sensor placement [54,55]. For successful adoption in clinical and research practice, IMU-based systems require rigorous validation before being integrated into motion analysis and rehabilitation settings. Ensuring consistent calibration, appropriate sensor placement, and accounting for inter-subject variability is critical.

Thus, the aim of this study was to validate a commercially available multi-sensor IMU-based system used in clinical practice against a gold-standard MCap system through lower limb, trunk, and upper limb motor tasks.

## 2. Materials and Methods

This prospective validation study aimed to assess and compare motion data recorded simultaneously by an IMU-based system and an optoelectronic motion capture system (MCap). The study was conducted at the “Luigi Divieti” Posture and Movement Analysis Laboratory (Department of Electronics, Information and Bioengineering (DEIB), Politecnico di Milano, Milano, Italy) in accordance with the Declaration of Helsinki and approved by the Ethics Committee of the Politecnico di Milano (Protocol No. 22/2021, 14 June 2021).

### 2.1. Participants

A total of 15 healthy participants (M/F: 6/9; age: 25.9 ± 3.8 years; body mass: 65.1 ± 14.3 kg; height: 170.0 ± 9.2 cm; BMI: 22.5 ± 3.4 kg/m^2^) were included in the study based on the following inclusion criteria: (i) age ≥ 18 years, (ii) absence of pain or musculoskeletal injuries within the 15 days prior to testing, (iii) no underlying musculoskeletal conditions that could affect test outcomes, and (iv) no regular participation in competitive or professional sports (>8 h/week of structured sport). Prior to participation, all volunteers received an explanation of the study’s objectives and provided informed written consent.

### 2.2. Equipment

Data were simultaneously collected from IMU-based and MCap systems.

The IMU-based system consisted of five inertial measurement units (Movella DOT, Enschede, The Netherlands) connected via Bluetooth Low Energy (BLE) to proprietary software (Euleria Lab, Euleria srl, Rovereto, Italy). Each IMU included a tri-axial accelerometer (±16 g full scale), a tri-axial gyroscope (±2000 deg/s full scale), and a tri-axial magnetometer (±8 Gauss full scale). Accelerometer and gyroscope signals were sampled at 800 Hz, whereas magnetometer signals were sampled at 60 Hz. A proprietary strapdown integration sensor fusion algorithm combined these data to output orientation quaternions at 60 Hz, as described in [56]. The IMUs were attached to participants’ bodies using non-invasive elastic bands to minimize skin motion artifacts.

The gold-standard MCap system consisted of eight infrared cameras (SMART DX 100, BTS-Bioengineering, Milan, Italy), providing accuracy of <0.2 mm over a 2 × 2 × 2 m volume, operating at 100 Hz.

To synchronize signals from the two systems, MCap data were resampled at 60 Hz to match the IMU output frequency using MATLAB (v.2023a MathWorks, Natick, MA, USA) *resample* function, which performs polyphase interpolation followed by anti-aliasing filtering.

### 2.3. Procedure

Participants were informed of the study’s purpose, and anthropometric measurements were recorded, including height, weight, leg length, knee diameter (distance between femoral condyles), ankle diameter (distance between malleoli), distance between the anterior iliac spines, and pelvis thickness.

IMUs and reflective markers were positioned following two different configurations based on the assessed tasks.

**Figure 1:**
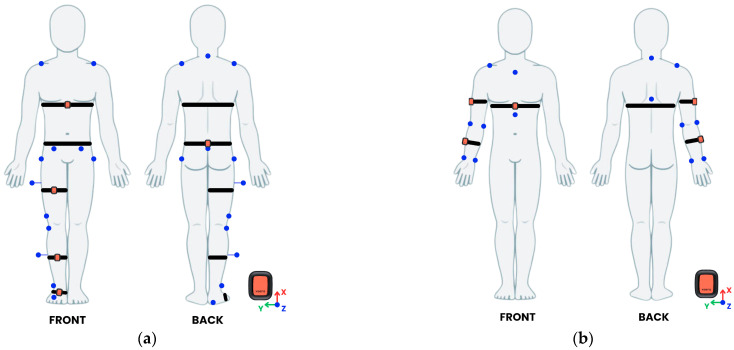
Reflective marker (in blue) and IMU sensor (in orange) configurations for the assessment of (**a**) lower limb and trunk tasks and (**b**) upper limb tasks.

For lower body and trunk motor tasks, five IMUs were mounted on the chest (below xiphoid process), pelvis (L5 vertebra level, midpoint between posterior superior iliac spines), anterior right thigh, anterior right shank and dorsal right foot. Fifteen reflective markers were placed on anatomical landmarks, according to a modified Davis protocol for the right side only (Figure 1a) [57].

For upper body tasks, three IMUs were mounted on the chest (below the xiphoid process), lateral right arm, and lateral right forearm. Nine reflective markers were placed at the anatomical landmarks displayed in Figure 1b [58].

After positioning IMUs and markers, both the IMU-based and the MCap systems were simultaneously calibrated. Participants assumed an anatomical N-pose calibration position: upright stance, feet parallel (about 20 cm apart), arms slightly abducted from the body, and palms facing forward. For the MCap system, calibration was performed to define participant anatomy and joint centers of rotation, as detailed in [57].

Following calibration, participants performed three repetitions of single-joint and multi-joint tasks, described sequentially in Table 1. To ensure consistency and minimize variability due to execution speed, participants were instructed to perform each movement at a controlled speed, reaching maximum joint excursion within approximately three seconds.

### 2.4. Data Processing

Raw IMU data were processed internally by Euleria Lab software, computing 3D joint angles (i.e., from quaternions preprocessed on-board by the Movella DOT proprietary sensor fusion algorithm [55]) through the processing pipeline disclosed in the following paragraphs. During the N-pose calibration, each sensor produced an initial calibration quaternion $q_{0}=(q_{w},q_{x},q_{y},q_{z})$ that mapped the global laboratory frame *G* to the sensor frame *S*. Each calibration quaternion was converted into the corresponding rotation matrix $R_{G}^{S}$, expressing the orientation S with respect to G (Equation (1)):

(1) $$R_{G}^{S}=\left[\begin{array}{ccc} 1-2\left(q_{y}^{2}+q_{z}^{2}\right) & 2({q_{x}q}_{y}-{q_{z}q}_{w}) & 2({q_{x}q}_{z}-{q_{y}q}_{w})\\ 2({q_{x}q}_{y}-{q_{z}q}_{w}) & 1-2\left(q_{x}^{2}+q_{z}^{2}\right) & 2({q_{y}q}_{z}-{q_{x}q}_{w})\\ 2({q_{x}q}_{z}-{q_{y}q}_{w}) & 2({q_{y}q}_{z}-{q_{x}q}_{w}) & 1-2\left(q_{x}^{2}+q_{y}^{2}\right) \end{array}\right]$$

For biomechanical consistency, the three sensor axes (X,Y,Z) had to be remapped (i.e., permuted and sign-flipped) to make *S* coincide with the conventional anatomical axes of the body segment to which the sensor is attached (anteroposterior, craniocaudal, and mediolateral, respectively). Applying this fixed permutation to $R_{G}^{S}$ yields the refined fixed axis-alignment rotation matrix that aligns the sensor axes to the anatomical axes. This refined rotation matrix was then reconverted into the quaternion $q_{0,ref}$, that represents the orientation of the sensor-segment assembly in the global frame at the calibration neutral pose. During motion, each IMU provided an instantaneous quaternion ($q_{RT}$) relative to the global frame. By combining $q_{RT}$ and $q_{0,ref}$ (Equation (2)), the orientation of the body segment (i.e., the rigid segment to which the sensor is affixed) with respect to its calibrated anatomical neutral position is obtained:

(2) $$q_{rel}=q_{RT}\;⨂\;{\left(q_{0,ref}\right)}^{-1}$$

where ⨂ denotes the Hamilton product, and $q^{-1}$ the quaternion conjugate. $q_{rel}$ describes how far and about which axis the current segment orientation has rotated away from the neutral pose, independently of the global laboratory coordinates.

For each anatomical joint, the joint motion is computed by obtaining joint quaternions ($q_{joint}$) as the relative rotation between the distal (*d*) and proximal (*p*) segments (Equation (3)):

(3) $$q_{joint}={\left(q_{rel,p}\right)}^{-1}\;⨂\;q_{rel,d}$$

The resulting joint quaternions $q_{joint}$ were decomposed using the ZYX Euler sequence to obtain clinically interpretable joint angles (flexion–extension, abduction–adduction, internal–external rotation).

MCap data were processed with SmartTracker and SmartAnalyzer (BTS Bioengineering, Milan, Italy). Data were tracked, interpolated, and filtered (5 Hz Butterworth low-pass filter) to obtain a 3D reconstruction of the coordinates of each marker. Custom routines extracted angular measures.

For lower body tasks, the limb rotation algorithm was based on Euler angles, and it defined joint movements in terms of flexion/extension, abduction/adduction, and internal/external rotation [57]. Trunk and pelvic angles were computed as absolute angles, while hip, knee, and ankle joint angles were expressed as relative angles. For the upper body, shoulder angles were defined as the angle between the humeral axis (acromion–lateral epicondyle) and thorax planes. Elbow angles were computed between humeral axis and forearm axis (lateral elbow–lateral wrist epicondyle).

Finally, ROM values for each task were calculated as the difference between the maximum and minimum joint angles during each repetition. A repetition was defined as a single execution of the task-specific movement, from the calibrated N-pose position to the point of maximal joint excursion and concluding upon the subject’s return to the initial position. Then, the corresponding ROM value for both the IMU-based and MCap systems was obtained as the average result of the three repeated movements.

### 2.5. Sample Size

The sample size for this study was determined by considering the specific population’s characteristics, feasibility constraints, and statistical requirements. A priori power analysis, conducted with G*Power, indicated that 15 participants would achieve 80% power at an alpha level of 0.05, assuming a large effect size (Cohen’s *d* = 0.8). This number of participants balanced practical considerations (e.g., participant availability) with the need for sufficient statistical sensitivity.

### 2.6. Statistical Analysis

Data normality was verified using the Shapiro–Wilk test for each task, and all the ROM values followed a normal distribution (*p* > 0.05). Consequently, mean and standard deviation (SD) were used for descriptive statistics, and parametric methods were employed [59,60]. To test systematic differences between the IMU-based and MCap system for each test, a paired samples *t*-test was performed, using α = 0.05 as the significance threshold. Effect sizes (Cohen’s *d*) were computed and classified as small (|*d*| < 0.2), moderate (|*d*| = 0.5), or large (|*d*| = 0.8) [61].

Accuracy was quantified by calculating the percentage of similarity (PoS, %) and the root mean square error (RMSE, °) as shown in Equations (4) and (5):

(4) $$PoS\;\left(\%\right)=1-\frac{\left|{ROM}_{IMU}-{ROM}_{MCap}\right|}{{ROM}_{MCap}}\times 100$$

(5) $$RMSE\;\left({}^{\circ}\right)=\sqrt{\frac{\sum_{i=1}^{N}{\left({ROM}_{IMU}\left(i\right)-{ROM}_{MCap}(i)\right)}^{2}}{N}}$$

Bland–Altman analyses were then conducted to assess agreement between the two measurement systems, yielding systematic bias and 95% limits of agreement (LoAs = bias ± 1.96 SD) for each task [62]. Pearson’s correlation coefficients (r) were also computed to evaluate the linear relationship between the two systems, with correlation strength defined as poor ($\left|r\right|<0.2$), fair ($\left|r\right|=0.3–0.5$), moderate ($\left|r\right|=0.6–0.7$), or strong ($\left|r\right|>0.8$) [63]. All statistical analyses were carried out using MATLAB (v.2023a MathWorks, Natick, MA, USA) and JASP (JASP Team 2023, Version 0.17.3).

## 3. Results

For clarity and ease of reference, the results are organized into four sub-sections based on body region and task complexity. The results are reported in tables in which each row reports mean (±SD) ROM values for both the IMU- and MCap-based systems, *p*-values and Cohen’s *d* from paired *t*-tests, accuracy metrics (PoS and RMSE), and Pearson’s correlation coefficients (r).

### 3.1. Lower Body and Trunk

The results for lower body and trunk single-joint tasks are summarized in Table 2.

The IMU system demonstrated excellent concurrent validity with the MCap system for all single-joint lower body and trunk tasks. No statistically significant differences were observed (*p* > 0.05), and the associated Cohen’s *d* values were uniformly small, indicating that any mean differences were trivial relative to the within-participant variability. Absolute agreement was likewise high (PoS > 98% and RMSE < 4.5°), confirming that the IMU estimates lay very close to the reference values. Correlation coefficients are notably high (*r* ≥ 0.89), indicating that the IMU measurements closely follow the same patterns as those captured by the MCap system. For the multi-joint tasks, the pattern was broadly similar. Both the hip and knee ROM during the squat showed no statistically significant differences (*p* ≥ 0.820, d ≤ 0.07) from the MCap system. High correlations (*r* ≥ 0.93) between IMU and MCap-based measurements during the squat were displayed, reinforcing the close alignment of motion patterns between the two systems. Hip ROM during the lunge exhibited a statistically significant difference (*p* = 0.034), accompanied by a moderate effect size (Cohen’s *d* = 0.661), slightly lower accuracy (PoS = 94.6%) and the largest RMSE of the set (6.9°). Despite this, the correlation for lunge hip ROM remains strong (*r* = 0.93), implying that the IMU-based system generally follows the same trend as the MCap reference. Overall, the IMU system reproduced lower body ROM with both high accuracy and strong linear association with respect to the reference system.

The agreement between the two systems for lower body and trunk movements was assessed through the Bland–Altman analysis, whose systematic bias and LoAs are reported in Table 3.

Bland–Altman analysis confirmed the close agreement observed in the paired comparisons. Systematic bias was negligible for every single-joint task, ranging from −1.0° (trunk flexion) to +1.2° (knee flexion), with corresponding LoA ranges within ±15°. For multi-joint tasks, bias remained small (−3.9° to 2.0°) in the squat and lunge knee, although the LoA ranges widened to about 30°, reflecting the increased kinematic variability inherent in compound movements. The wider differences were reported in the hip ROM during lunges, where the IMU underestimated the optical measurement by −3.9° on average and displayed an asymmetric LoA (−15.0° to +7.6°). This pattern echoes the significant paired-test result and suggests a modest but systematic under-reading for that specific motion.

### 3.2. Upper Body

The results for upper body tasks are summarized in Table 4.

For single-joint tasks, the IMU and MCap systems show no statistically significant differences (*p* ≥ 0.30, d ≤ 0.32) and high accuracy (PoS > 97%, RMSE ≈ 3.7–6.6°). Pearson’s *r* reveals strong correlation for the shoulder abduction task (*r* = 0.92), but moderate correlation for shoulder and elbow flexion (*r* = 0.77 and *r* = 0.80, respectively). Both the shoulder and elbow angles during the overhead press task displayed excellent agreement with non-significant differences (*p* ≥ 0.59) and negligible effect sizes (*d* ≤ 0.16). Accuracy and correlation results are comparable to the ones for the single-joint tasks (PE ≥ 98.5%, RMSE < 7°, *r* > 0.93). These findings indicate that, despite the increased kinematic complexity of a coordinated press, the IMU system reproduces reference measurements with both high absolute precision and close tracking of inter-individual variability.

The agreement between the two systems for upper body movements was assessed through the Bland–Altman analysis, whose systematic bias and LoAs are reported in Table 5.

The results from the Bland–Altman analysis reported a systematic bias within ±2° with an overall overestimation of the ROM from IMU-based system with respect to the MCap. The task complexity (single-joint or multi-joint task) does not impact systematic bias and LoAs. The task showing the narrowest LoA range is the shoulder flexion (14.6°), while multi-joint tasks show wider LoAs (~27°).

## 4. Discussion

The present study aimed to evaluate the concurrent validity of a commercially available multi-sensor IMU-based motion-capture system against a laboratory-grade optoelectronic reference system during a comprehensive battery of single- and multi-joint movements involving the lower limbs, trunk, and upper limbs. Overall, the findings align with the previous literature, demonstrating that the IMU-based system reproduces joint-specific ROM measurements with accuracy and agreement comparable to the reference system.

For all single-joint lower body and trunk movements, mean biases were ≤1.2°, PoS values exceeded 98%, and RMSE remained below 4.5°. These results indicate excellent absolute agreement and are consistent with previous validations reporting RMSE values below 5° for lower limb joints when IMUs were benchmarked against marker-based systems [42,48,49]. Correlation coefficients were uniformly strong (*r* ≥ 0.89), underscoring that inter-individual variability captured by the IMU system closely mirrors that of the gold standard.

During compound movements, the IMU showed high accuracy for squat-related hip and knee ROM (RMSE ≤ 7.1°) and retained strong correlations (*r* ≥ 0.93). The only statistically significant discrepancy emerged for hip flexion during the lunge, where the IMU underestimated ROM by 3.9° on average and showed a moderate effect size (*d* = 0.661). This under-reading likely reflects the increased pelvic tilt and multi-planar motion inherent in the lunge, which may exacerbate soft-tissue artefacts and violate the single rigid segment assumption embedded in the sensor-fusion algorithm [39,40,41]. Nevertheless, even for this task, the error magnitude (RMSE = 6.9°) remained within clinically acceptable limits [13].

In single-joint upper limb tasks, the IMU reproduced RMSE values between 3.7° and 6.6° and no significant mean differences with respect to the MCap system. While correlations were variable (*r* = 0.77–0.92), the strength of association was still sufficient for clinical trend monitoring and is comparable to earlier reports on humerothoracic joint angles (*r* ≥ 0.80, RMSE < 6°) [42,43,44,45]. During the multi-joint movements, the IMU reliably tracked coordinated upper body motions despite their larger excursion and speed, achieving strong correlations (*r* = 0.93–0.96) and RMSE values < 6.7°. The results indicate that the IMU system can reliably track coordinated multi-joint upper body motions despite their larger excursion and speed. These upper limb outcomes again align with published Pearson’s *r* and RMSE benchmarks for IMU-based systems [47,48].

Several factors likely contributed to the high level of agreement observed. From a methodological point of view, the adoption of a standardized N-pose calibration protocol, combined with careful alignment of sensor axes to their corresponding anatomical axes, helped reduce the likelihood of initial orientation errors [23,24,25,26]. Secure sensor positioning on prominent body landmarks minimized axis misalignment and crosstalk—common sources of error in IMU-based measurements. By minimizing systematic offsets at the outset, the accuracy of subsequent joint angle estimations was enhanced.

With respect to task selection, this research included both single-joint and compound movements to fully assess the performance of the IMU-based system. Single-joint tasks (e.g., hip, knee, and ankle flexion/extension) are ideal for assessing the sensors’ ability to capture specific joint movements with minimal interference from other body parts [13]. In contrast, compound movements (e.g., squats and lunges) involve multiple joints and body segments, mimicking more complex functional motions seen in daily activities and rehabilitation exercises. Moreover, by imposing a controlled-speed motion, the study reduced potential issues like sensor drift that can distort data during fast movements [40]. This diversified yet speed-controlled task set therefore enhances the clinical relevance of the results for rehabilitation settings.

Taken together, these results suggest that the tested IMU configuration delivers ROM estimates that are interchangeable with a laboratory-grade MCap system for most tasks. Given their portability, affordability, and independence from dedicated spaces or cameras [21,22], IMUs offer clear logistical advantages for both in-clinic and at-home rehabilitation programs, where frequent assessments and real-time feedback are desirable.

Despite the highlighted strengths, some limitations warrant caution.

Although the sample size was sufficient based on the power analysis, the restricted number and age range of participants may limit the generalizability of the findings. Indeed, subjects affected by movement disorders (e.g., orthopedic and post-stroke patients) who may exhibit different biomechanical patterns should be included in future studies to understand the sensors’ accuracy under altered motor movements. Moreover, the current study was conducted in a controlled laboratory environment, ensuring experimental consistency but inherently limiting its generalizability to real-world clinical environments characterized by higher variability. External factors such as ferromagnetic disturbances—common in clinical settings—or inaccuracies due to sensor misplacement may negatively impact IMU performance [23,24,48]. To mitigate these magnetic disturbances, several strategies can be considered. These include adopting magnetometer-free sensor fusion algorithms, employing adaptive filtering methods capable of detecting and compensating for magnetic anomalies in real-time, and performing initial environmental assessments to identify zones of elevated magnetic interference. Furthermore, practical measures such as periodic on-site calibration procedures, adherence to clear sensor placement guidelines (e.g., avoiding proximity to ferromagnetic materials), and using stationary reference magnetometers to monitor environmental magnetic fluctuations can significantly enhance the robustness, accuracy, and clinical reliability of IMU systems in clinical contexts.

The restricted sensor configuration may have introduced limitations in capturing complex body movements. The study employed five sensors for lower body tasks and three for upper body tasks, in accordance with the manufacturer’s standard clinical workflow. While this sensor placement strategy is practical and aligned with existing protocols, it may not fully account for all sources of segment-interaction errors, particularly during multi-planar motions. In addition, the accuracy reported in the present study applies to the specific hardware–firmware evaluated (i.e., the Movella DOT sensors running the proprietary Kalman filter, as described in [56]). Although this filter architecture is representative of many state-of-the-art algorithms, its proprietary gain tuning is not publicly disclosed, and performance may therefore vary when alternative IMU modules or custom fusion pipelines are employed. Consequently, any implementation that departs from the pipeline tested in this study should be preceded by a focused concurrent validity assessment to verify that orientation error and drift remain within clinically acceptable limits.

Another methodological limitation concerns the use of peak-to-peak ROM as the primary outcome measure. While ROM is a widely adopted and clinically meaningful parameter, it provides only a summary of the joint excursion and does not capture the temporal characteristics of movement. As such, it overlooks potentially important differences in movement patterns throughout the execution of a task. Incorporating waveform-level analyses (e.g., statistical parametric mapping (SPM)) would allow for a more detailed comparison between IMU and MCap systems, offering insights into the timing and coordination of joint kinematics during dynamic or rehabilitative movements [64].

Finally, the evaluation of sensor drift was limited to short-duration trials. Each recording captured discrete trials of movement, which may not fully capture the cumulative integration errors that can accrue over prolonged monitoring periods. Drift, a known limitation of IMU-based systems, is often negligible in short bouts but may become clinically significant during continuous or long-term use [24,25]. Longer duration studies are thus essential to characterize drift behaviors under sustained operation and to inform strategies for mitigating its impact in real-world applications.

Future investigations should extend the validation of the IMU-based system to pathological cohorts in which joint kinematic patterns, soft-tissue artefacts and the presence of assistive devices may challenge inertial sensing. Task-specific protocols (e.g., over-ground walking at self-selected and fast speeds, sit-to-stand transitions, and obstacle negotiation) are recommended to capture clinically relevant movement deviations in populations affected by hemiparetic gait after stroke, Parkinson’s disease, cerebral palsy, and in post-arthroplasty patients. In parallel, dynamic activities such as running, change of direction, and landing should be explored to characterize the system’s performance under high acceleration and impact-induced vibrations.

From an engineering perspective, developing adaptive magnetometer-rejection filters could reduce interference from real-environment setting magnetic fields to improve consistency and scalability across clinical settings. Finally, prospective field studies should embed the system in practical scenarios—outpatient ROM screening, bedside assessment on rehabilitation wards, and unsupervised telerehabilitation—to determine usability, reliability, and responsiveness to change during multi-week recovery programs. Such comprehensive real-world validation will be essential to confirm the scalability of IMU-based motion analysis beyond the controlled laboratory settings.

## 5. Conclusions

The present validation study demonstrated that a commercially available multi-sensor IMU system can replicate the joint-specific range-of-motion measurements of a laboratory-grade optoelectronic reference across a wide variety of movements involving the lower limbs, trunk, and upper limbs. Bland–Altman analysis revealed trivial systematic biases and limits of agreement no wider than ±15°, which further supports the interchangeability of the two systems for most functional tasks.

Methodological choices—such as a standardized N-pose calibration, precise sensor alignment to anatomical axes, and secure fixation on bony landmarks—likely minimized orientation error, crosstalk, and soft-tissue artefact, thereby underpinning the high level of agreement observed. The evidence indicates that the ROM estimates from IMU sensors are both statistically and clinically equivalent to those obtained with optical motion capture, while conferring practical advantages in cost, portability, and set-up flexibility.

Future work should extend validation to pathological populations, incorporate waveform-level analyses to capture temporal kinematic characteristics, and examine long-duration trials to quantify drift.

## Figures and Tables

**Table 1:** Description of the tasks performed in their order of execution.

Body District and Complexity	Task	Description
Lower body and trunk Single-joint	Hip Abd	Standing on the left leg, raise the right leg laterally away from the body at maximum ROM, keeping the pelvis stable.
	Hip Flex	Standing on the left leg, raise the right leg forward by flexing at the hip at maximum ROM.
	Knee Flex	Seated on a box with legs hanging, bend and extend the right knee at maximum ROM.
	Ankle Flex	Seated on a box with legs hanging, perform plantar- and dorsi-flexion of the right ankle at maximum ROM.
	Trunk Flex	Standing upright, perform trunk flexion by hinging at the hips at approximately 45°.
Lower body Multi-joint	Squat	With feet shoulder-width apart, lower the body by bending both knees and hips while keeping a straight back, then return to standing.
	Lunge	Standing upright, step forward with the right leg and lower the body until both knees form roughly 90° angles (with the front knee aligned over the ankle), then push off to return to the starting position.
Upper body Single-joint	Shoulder Flex	While standing with the arm by the side, lift the arm forward and upward at maximum ROM.
	Shoulder Abd	While standing with the arm by the side, lift the arm laterally away from the body at maximum ROM.
	Elbow Flex	While standing with the arm by the side, bend the elbow to bring the forearm toward the upper arm at maximum ROM while keeping the wrist neutral.
Upper body Multi-joint	Overhead Press	While standing, hold a stick with hands shoulder-width apart and push the stick upward from shoulder level until full arm extension is reached.

Abd = Abduction, Flex = Flexion.

**Table 2:** IMU-based and MCap-based ROM values (mean ± SD), *t*-test results (*p*-values, Cohen’s *d*), accuracy metrics (PoS, RMSE), and Pearson’s correlation coefficients (*r*) for lower body and trunk tasks.

Complexity	Task	ROM (Mean ± SD)		*t*-Test		Accuracy		Correlation
		IMU (°)	MCap (°)	*p*-Values	Cohen’s *d*	PoS (%)	RMSE (°)	*r*
Single-joint	Hip Abd	27.19 ± 7.60	26.93 ± 8.02	0.795	0.071	99.0	3.6	0.89 *
	Hip Flex	44.51 ± 17.20	45.09 ± 18.64	0.563	−0.172	98.7	3.1	0.99 *
	Knee Flex	59.11 ± 15.75	60.33 ± 16.59	0.098	−0.498	98.0	2.7	0.99 *
	Ankle Flex	43.13 ± 17.66	42.56 ± 17.81	0.660	0.129	98.7	4.2	0.97 *
	Trunk Flex	54.02 ± 11.60	53.04 ± 13.67	0.373	0.257	98.1	3.7	0.97 *
Multi-joint	Squat—Hip Flex	73.56 ± 19.40	73.07 ± 15.59	0.820	0.067	99.3	6.2	0.93 *
	Squat—Knee Flex	70.75 ± 20.16	70.58 ± 16.05	0.938	0.023	99.8	7.1	0.94 *
	Lunge—Hip Flex	76.42 ± 14.13	72.53 ± 10.36	0.034 *	0.661	94.6	6.9	0.93 *
	Lunge—Knee Flex	80.51 ± 12.62	82.51 ± 13.72	0.177	−0.398	97.6	2.2	0.93 *

* indicates significant *p*-values. Abd = Abduction, Flex = Flexion.

**Table 3:** Bland–Altman analysis results for lower body and trunk tasks.

Complexity	Task	Systematic Bias (LoAs) (°)
Single-joint	Hip Abd	−0.3 (−7.6 to 7.0)
	Hip Flex	0.6 (−6.0 to 7.2)
	Knee Flex	1.2 (−3.6 to 6.0)
	Ankle Flex	−0.6 (−9.2 to 8.1)
	Trunk Flex	−1.0 (−8.5 to 6.5)
Multi-joint	Squat—Hip Flex	−0.5 (−15.0 to 14.0)
	Squat—Knee Flex	−0.2 (−15.0 to 14.0)
	Lunge—Hip Flex	−3.9 (−15.0 to 7.6)
	Lunge—Knee Flex	2.0 (−7.9 to 12.0)

Abd = Abduction, Flex = Flexion.

**Table 4:** IMU-based and MCap-based ROM values (mean ± SD), *t*-test results (*p*-values, Cohen’s *d*), accuracy metrics (PoS, RMSE), and Pearson’s correlation coefficients (*r*) for upper body tasks.

Complexity	Task	ROM (Mean ± SD)		*t*-Test		Accuracy		Correlation
		IMU (°)	MCap (°)	*p*-Values	Cohen’s *d*	PoS (%)	RMSE (°)	*r*
Single-joint	Shoulder Abd	78.36 ± 8.83	79.39 ± 9.19	0.339	−0.276	98.7	3.7	0.92 *
	Shoulder Flex	75.58 ± 9.87	74.43 ± 5.07	0.570	0.169	98.5	6.6	0.77 *
	Elbow Flex	88.36 ± 10.84	86.28 ± 9.91	0.297	0.316	97.6	6.6	0.80 *
Multi-joint	Overhead press—Shoulder Ext	74.97 ± 17.55	73.87 ± 18.78	0.587	0.162	98.5	6.7	0.93 *
	Overhead press—Elbow Ext	69.86 ± 19.34	69.51 ± 16.81	0.828	0.062	99.5	5.5	0.96 *

* indicates significant *p*-values. Abd = Abduction, Flex = Flexion, Ext = Extension.

**Table 5:** Bland–Altman analysis results for upper body tasks.

Complexity	Task	Systematic Bias (LoAs) (°)
Single-joint	Shoulder Abd	−1.1 (−14.0 to 12.0)
	Shoulder Flex	1.0 (−6.3 to 8.3)
	Elbow Flex	−2.1 (−15.0 to 11.0)
Multi-joint	Overhead press—Shoulder Ext	−1.1 (−15.0 to 12.0)
	Overhead press—Elbow Ext	−0.4 (−12.0 to 11.0)

Abd = Abduction, Flex = Flexion, Ext = Extension.

## Data Availability

The raw data supporting the conclusions of this article will be made available by the authors upon request.

## Abbreviations

The following abbreviations are used in this manuscript:

IMU	Inertial Measurement Unit
MCap	Motion Capture
ROM	Range Of Motion
Abd	Abduction
Flex	Flexion
Ext	Extension
RMSE	Root Mean Square Error
PoS	Percentage of Similarity
LoAs	Limits of Agreement

## References

[1] Hochstenbach J. (2000). Rehabilitation Is More than Functional Recovery. Disabil. Rehabil..

[2] Andersen L.L., Saervoll C.A., Mortensen O.S., Poulsen O.M., Hannerz H., Zebis M.K. (2011). Effectiveness of Small Daily Amounts of Progressive Resistance Training for Frequent Neck/Shoulder Pain: Randomised Controlled Trial. Pain.

[3] Latash M.L., Anson J.G. (2006). Synergies in Health and Disease: Relations to Adaptive Changes in Motor Coordination. Phys. Ther..

[4] Veldema J., Jansen P. (2020). Resistance Training in Stroke Rehabilitation: Systematic Review and Meta-Analysis. Clin. Rehabil..

[5] Nussbaumer S., Leunig M., Glatthorn J.F., Stauffacher S., Gerber H., Maffiuletti N.A. (2010). Validity and Test-Retest Reliability of Manual Goniometers for Measuring Passive Hip Range of Motion in Femoroacetabular Impingement Patients. BMC Musculoskelet. Disord..

[6] Muir S.W., Corea C.L., Beaupre L. (2010). Evaluating Change in Clinical Status: Reliability and Measures of Agreement for the Assessment of Glenohumeral Range of Motion. N. Am. J. Sports Phys. Ther..

[7] Walmsley C.P., Williams S.A., Grisbrook T., Elliott C., Imms C., Campbell A. (2018). Measurement of Upper Limb Range of Motion Using Wearable Sensors: A Systematic Review. Sports Med.-Open.

[8] Boone D.C., Azen S.P., Lin C.M., Spence C., Baron C., Lee L. (1978). Reliability of Goniometric Measurements. Phys. Ther..

[9] Lim C.C., Affandi M., Basah S.N., Din M.Y. (2018). Evaluating Lower Limb Joint Flexion by Computerized Visual Tracking System and Compared with Electrogoniometer and Universal Goniometer. J. Telecommun. Electron. Comput. Eng..

[10] McGinley J.L., Baker R., Wolfe R., Morris M.E. (2009). The Reliability of Three-Dimensional Kinematic Gait Measurements: A Systematic Review. Gait Posture.

[11] Colyer S.L., Evans M., Cosker D.P., Salo A.I.T. (2018). A Review of the Evolution of Vision-Based Motion Analysis and the Integration of Advanced Computer Vision Methods Towards Developing a Markerless System. Sports Med.-Open.

[12] Roggio F., Ravalli S., Maugeri G., Bianco A., Palma A., Di Rosa M., Musumeci G. (2021). Technological Advancements in the Analysis of Human Motion and Posture Management through Digital Devices. World J. Orthop..

[13] Poitras I., Dupuis F., Bielmann M., Campeau-Lecours A., Mercier C., Bouyer L.J., Roy J.-S. (2019). Validity and Reliability of Wearable Sensors for Joint Angle Estimation: A Systematic Review. Sensors.

[14] Adesida Y., Papi E., McGregor A.H. (2019). Exploring the Role of Wearable Technology in Sport Kinematics and Kinetics: A Systematic Review. Sensors.

[15] Gu C., Lin W., He X., Zhang L., Zhang M. (2023). IMU-Based Motion Capture System for Rehabilitation Applications: A Systematic Review. Biomim. Intell. Robot..

[16] Arlotti J.S., Carroll W.O., Afifi Y., Talegaonkar P., Albuquerque L., Burch V., Ball J.E., Chander H., Petway A. (2022). Benefits of IMU-Based Wearables in Sports Medicine: Narrative Review. Int. J. Kinesiol. Sports.

[17] Horak F., King L., Mancini M. (2015). Role of Body-Worn Movement Monitor Technology for Balance and Gait Rehabilitation. Phys. Ther..

[18] Seel T., Raisch J., Schauer T. (2014). IMU-Based Joint Angle Measurement for Gait Analysis. Sensors.

[19] Teufl W., Miezal M., Taetz B., Fröhlich M., Bleser G. (2018). Validity, Test-Retest Reliability and Long-Term Stability of Magnetometer Free Inertial Sensor Based 3D Joint Kinematics. Sensors.

[20] Mundt M., Koeppe A., David S., Witter T., Bamer F., Potthast W., Markert B. (2020). Estimation of Gait Mechanics Based on Simulated and Measured IMU Data Using an Artificial Neural Network. Front. Bioeng. Biotechnol..

[21] Komaris D.-S., Tarfali G., O’Flynn B., Tedesco S. (2022). Unsupervised IMU-Based Evaluation of at-Home Exercise Programmes: A Feasibility Study. BMC Sports Sci. Med. Rehabil..

[22] Cho Y.-S., Jang S.-H., Cho J.-S., Kim M.-J., Lee H.D., Lee S.Y., Moon S.-B. (2018). Evaluation of Validity and Reliability of Inertial Measurement Unit-Based Gait Analysis Systems. Ann. Rehabil. Med..

[23] De Vries W.H.K., Veeger H.E.J., Baten C.T.M., Van Der Helm F.C.T. (2009). Magnetic Distortion in Motion Labs, Implications for Validating Inertial Magnetic Sensors. Gait Posture.

[24] Camomilla V., Bergamini E., Fantozzi S., Vannozzi G. (2018). Trends Supporting the In-Field Use of Wearable Inertial Sensors for Sport Performance Evaluation: A Systematic Review. Sensors.

[25] Filippeschi A., Schmitz N., Miezal M., Bleser G., Ruffaldi E., Stricker D. (2017). Survey of Motion Tracking Methods Based on Inertial Sensors: A Focus on Upper Limb Human Motion. Sensors.

[26] Eichelberger P., Ferraro M., Minder U., Denton T., Blasimann A., Krause F., Baur H. (2016). Analysis of Accuracy in Optical Motion Capture—A Protocol for Laboratory Setup Evaluation. J. Biomech..

[27] Piche E., Guilbot M., Chorin F., Guerin O., Zory R., Gerus P. (2022). Validity and Repeatability of a New Inertial Measurement Unit System for Gait Analysis on Kinematic Parameters: Comparison with an Optoelectronic System. Measurement.

[28] Dorschky E., Nitschke M., Seifer A.-K., van den Bogert A.J., Eskofier B.M. (2019). Estimation of Gait Kinematics and Kinetics from Inertial Sensor Data Using Optimal Control of Musculoskeletal Models. J. Biomech..

[29] Zhao Y., Liu Y., Li J., Wang X., Yang R., Lian C., Shan P., Wang Y., Zhan Z., Fu C. (2024). Global Joint Information Extraction Convolution Neural Network for Parkinson’s Disease Diagnosis. Expert. Syst. Appl..

[30] Camuncoli F., Barni L., Nutarelli S., Rocchi J.E., Barcillesi M., Di Dio I., Sambruni A., Galli M. (2022). Validity of the Baiobit Inertial Measurements Unit for the Assessment of Vertical Double- and Single-Leg Countermovement Jumps in Athletes. Int. J. Environ. Res. Public Health.

[31] Toft Nielsen E., Jørgensen P.B., Mechlenburg I., Sørensen H. (2019). Validation of an Inertial Measurement Unit to Determine Countermovement Jump Height. Asia-Pac. J. Sports Med. Arthrosc. Rehabil. Technol..

[32] Marković S., Dopsaj M., Tomažič S., Kos A., Nedeljković A., Umek A. (2021). Can IMU Provide an Accurate Vertical Jump Height Estimate?. Appl. Sci..

[33] Blandeau M., Guichard R., Hubaut R., Leteneur S. (2023). IMU Positioning Affects Range of Motion Measurement During Squat Motion Analysis. J. Biomech..

[34] Whelan D., O’Reilly M., Huang B., Giggins O., Kechadi T., Caulfield B. Leveraging IMU Data for Accurate Exercise Performance Classification and Musculoskeletal Injury Risk Screening. Proceedings of the 2016 38th Annual International Conference of the IEEE Engineering in Medicine and Biology Society (EMBC).

[35] Kianifar R., Joukov V., Lee A., Raina S., Kulić D. (2019). Inertial Measurement Unit-Based Pose Estimation: Analyzing and Reducing Sensitivity to Sensor Placement and Body Measures. J. Rehabil. Assist. Technol. Eng..

[36] Clemente F.M., Akyildiz Z., Pino-Ortega J., Rico-González M. (2021). Validity and Reliability of the Inertial Measurement Unit for Barbell Velocity Assessments: A Systematic Review. Sensors.

[37] Khuyagbaatar B., Tumurbaatar M., Tsenkherjav K., Purevsuren T., Shambaljamts T., Kim K., Danjkhuu T., Danaa G., Hyuk Kim Y. (2024). Kinematic Comparison of Snatch and Clean Lifts in Weightlifters Using Wearable Inertial Measurement Unit Sensors. Phys. Act. Health.

[38] O’Reilly M.A., Whelan D.F., Ward T.E., Delahunt E., Caulfield B.M. (2017). Classification of Deadlift Biomechanics with Wearable Inertial Measurement Units. J. Biomech..

[39] Teufl W., Miezal M., Taetz B., Fröhlich M., Bleser G. (2019). Validity of Inertial Sensor Based 3D Joint Kinematics of Static and Dynamic Sport and Physiotherapy Specific Movements. PLoS ONE.

[40] Robert-Lachaine X., Mecheri H., Larue C., Plamondon A. (2017). Validation of Inertial Measurement Units with an Optoelectronic System for Whole-Body Motion Analysis. Med. Biol. Eng. Comput..

[41] Unger T., De Sousa Ribeiro R., Mokni M., Weikert T., Pohl J., Schwarz A., Held J.P.O., Sauerzopf L., Kühnis B., Gavagnin E. (2024). Upper Limb Movement Quality Measures: Comparing IMUs and Optical Motion Capture in Stroke Patients Performing a Drinking Task. Front. Digit. Health.

[42] Cutti A.G., Giovanardi A., Rocchi L., Davalli A., Sacchetti R. (2008). Ambulatory Measurement of Shoulder and Elbow Kinematics through Inertial and Magnetic Sensors. Med. Bio Eng. Comput..

[43] Parel I., Cutti A.G., Kraszewski A., Verni G., Hillstrom H., Kontaxis A. (2014). Intra-Protocol Repeatability and Inter-Protocol Agreement for the Analysis of Scapulo-Humeral Coordination. Med. Biol. Eng. Comput..

[44] Friesen K.B., Wu L.Z., Waslen A., Lang A.E. (2023). Defining Repeatability for Scapulothoracic and Thoracohumeral Motion during the Novel Work-Related Activities and Functional Task (WRAFT) Protocol. J. Biomech..

[45] Fang Z., Woodford S., Senanayake D., Ackland D. (2023). Conversion of Upper-Limb Inertial Measurement Unit Data to Joint Angles: A Systematic Review. Sensors.

[46] Morrow M.M.B., Lowndes B., Fortune E., Kaufman K., Hallbeck M. (2017). Validation of Inertial Measurement Units for Upper Body Kinematics. J. Appl. Biomech..

[47] Li J., Qiu F., Gan L., Chou L.-S. (2024). Concurrent Validity of Inertial Measurement Units in Range of Motion Measurements of Upper Extremity: A Systematic Review and Meta-Analysis. Wearable Technol..

[48] Cerfoglio S., Capodaglio P., Rossi P., Conforti I., D’Angeli V., Milani E., Galli M., Cimolin V. (2023). Evaluation of Upper Body and Lower Limbs Kinematics through an IMU-Based Medical System: A Comparative Study with the Optoelectronic System. Sensors.

[49] Leardini A., Lullini G., Giannini S., Berti L., Ortolani M., Caravaggi P. (2014). Validation of the Angular Measurements of a New Inertial-Measurement-Unit Based Rehabilitation System: Comparison with State-of-the-Art Gait Analysis. J. Neuroeng. Rehabil..

[50] Felius R.A.W., Geerars M., Bruijn S.M., Wouda N.C., Van Dieën J.H., Punt M. (2022). Reliability of IMU-Based Balance Assessment in Clinical Stroke Rehabilitation. Gait Posture.

[51] Pan H., Wang H., Li D., Zhu K., Gao Y., Yin R., Shull P.B. (2024). Automated, IMU-Based Spine Angle Estimation and IMU Location Identification for Telerehabilitation. J. Neuroeng. Rehabil..

[52] Lobo P., Morais P., Murray P., Vilaça J.L. (2024). Trends and Innovations in Wearable Technology for Motor Rehabilitation, Prediction, and Monitoring: A Comprehensive Review. Sensors.

[53] Ettefagh A., Roshan Fekr A. (2024). Technological Advances in Lower-Limb Tele-Rehabilitation: A Review of Literature. J. Rehabil. Assist. Technol. Eng..

[54] Al-Amri M., Nicholas K., Button K., Sparkes V., Sheeran L., Davies J.L. (2018). Inertial Measurement Units for Clinical Movement Analysis: Reliability and Concurrent Validity. Sensors.

[55] Niswander W., Wang W., Kontson K. (2020). Optimization of IMU Sensor Placement for the Measurement of Lower Limb Joint Kinematics. Sensors.

[56] Paulich M., Schepers M., Rudigkeit N., Bellusci G. (2018). Xsens MTw Awinda: Miniature Wireless Inertial-Magnetic Motion Tracker for Highly Accurate 3D Kinematic Applications.

[57] Davis R.B., Õunpuu S., Tyburski D., Gage J.R. (1991). A Gait Analysis Data Collection and Reduction Technique. Hum. Mov. Sci..

[58] Goreham J.A., MacLean K.F.E., Ladouceur M. (2022). The Validation of a Low-Cost Inertial Measurement Unit System to Quantify Simple and Complex Upper-Limb Joint Angles. J. Biomech..

[59] Mishra P., Pandey C.M., Singh U., Gupta A., Sahu C., Keshri A. (2019). Descriptive Statistics and Normality Tests for Statistical Data. Ann. Card. Anaesth..

[60] Ghasemi A., Zahediasl S. (2012). Normality Tests for Statistical Analysis: A Guide for Non-Statisticians. Int. J. Endocrinol. Metab..

[61] Cohen J. (1988). Statistical Power Analysis for the Behavioral Sciences.

[62] Bland J.M., Altman D.G. (1986). Statistical Methods for Assessing Agreement Between Two Methods of Clinical Measurement. Lancet.

[63] Akoglu H. (2018). User’s Guide to Correlation Coefficients. Turk. J. Emerg. Med..

[64] Bonfiglio A., Petruccelli C., Villa G., Bongers R.M., Farella E., Kondylakis H., Triantafyllidis A. (2025). Preliminary Validation of an IMU-Based Physiotherapy Assessment System for the Lower Extremities. Pervasive Computing Technologies for Healthcare, Proceedings of the 18th EAI International Conference, PervasiveHealth 2024, Heraklion, Greece, 17–18 September 2024.

